# Evaluation of the Impact of Calcium Silicate-Based Sealer Insertion Technique on Root Canal Obturation Quality: A Micro-Computed Tomography Study

**DOI:** 10.3390/bioengineering10111331

**Published:** 2023-11-19

**Authors:** Germain Sfeir, Frédéric Bukiet, Marc Krikor Kaloustian, Naji Kharouf, Lotfi Slimani, Baptiste Casel, Carla Zogheib

**Affiliations:** 1 Department of Endodontics, Faculty of Dental Medicine, Saint Joseph University of Beirut, Beirut 17-5208, Lebanon; germainsfeir@gmail.com (G.S.); mkaloustian75@gmail.com (M.K.K.); zogheibcarla@gmail.com (C.Z.); 2 Assistance Publique des Hôpitaux de Marseille, 13005 France, Aix Marseille Univ, CNRS, ISM, Inst Movement Sci, 13288 Marseille, France; frederic.bukiet@univ-amu.fr; 3 Department of Biomaterials and Bioengineering, INSERM UMR_S 1121, 67000 Strasbourg, France; 4 Department of Endodontics, Faculty of Dental Medicine, Strasbourg University, 67000 Strasbourg, France; 5 Plateforme Imageries du Vivant, Faculté de Chirurgie Dentaire, Université de Paris, 92120 Montrouge, France

**Keywords:** calcium silicate-based sealer, endodontics, endodontic sealers, micro-computed tomography, obturation, root canal therapy

## Abstract

Background: Calcium silicate-based sealers have gained in popularity over time due to their physicochemical/biological properties and their possible use with single-cone obturation. The single cone technique is a sealer-based obturation and there is still a knowledge gap regarding the potential impact of the sealer insertion method on the root canal-filling quality. Therefore, the aim of this micro-CT study was to assess the impact of the calcium silicate-based sealer insertion technique on void occurrence and on the sealer extrusion following single-cone obturation. Methods: Thirty-six single-rooted mandibular premolars with one canal were shaped with Reciproc^®^ R25 (VDW, Munich, Germany) then divided randomly into four groups of nine canals, each depending on the TotalFill^®^ BC Sealer insertion technique used with single cone obturation: injection in the coronal two-thirds (group A); injection in the coronal two-thirds followed by direct sonic activation (group B); injection in the coronal two-thirds followed by indirect ultrasonic activation on tweezers (group C); sealer applied only on the master-cone (control group D). Samples were then scanned using micro-CT for voids and sealer extrusion calculation. Data were statistically analyzed using v.26 IBM; Results: No statistically significant differences were noted between the four groups in terms of voids; nevertheless, sonic activation (group B) followed by ultrasonic activation on the tweezers (group C) showed the best results (*p* = 0.066). Group D showed significantly less sealer extrusion when compared with group C (*p* = 0.044), with no statistically significant differences between groups D, A and B (*p* > 0.05). Conclusions: Despite no significant differences observed between the different sealer placement techniques, the use of sonic and ultrasonic activation might be promising to reduce void occurrence. Further investigations are needed to demonstrate the potential benefit of calcium silicate-based sealer activation especially in wide and oval root canals in order to improve the quality of the single-cone obturation.

## 1. Introduction

The long-term success of endodontic treatment depends on various factors including the quality of a tridimensional and hermetic root canal filling [1,2] which aims to minimize the risk of bacterial reinfection and promote periapical healing [3]. The significance of achieving an optimal obturation, with the fewest voids possible should not be underestimated. To date, sterilization of the root canal space has been demonstrated to be challenging, even impossible [4,5]. Therefore, voids in a filled root canal space should be considered as potential niches for residual microorganisms which may regrow overtime leading to jeopardize the endodontic outcome [6,7].

The emergence of calcium silicate-based sealers (CSBS) in the last few decades has been considered a paradigm shift considering their physicochemical and biological properties but also taking into account their possible combination with the single cone (SC) technique and overall the concept of hydraulic condensation [8,9]. For these reasons, these sealers have grown in popularity, and have been the subject of numerous investigations [8,10,11]. However, it is well known that the SC technique may induce a higher ratio of voids when compared to thermoplasticized gutta-percha obturation techniques, especially in oval root canals [8,9,10,11,12]. The SC technique is a sealer-based obturation concept, where the emphasis is put more on the sealer than on the gutta-percha [13]. Indeed, in the presence of CSBS, considering their good dimensional stability, higher film thickness and interactivity with dentin, the concept of SC was updated. However, the latter presents some limitations and induces a greater void occurrence [14,15]. Another factor involved in void occurrence might be the type of sealer insertion technique, especially in the case of sealer-based obturation, also depending on the root canal anatomy. In addition, premixed CSBS sealers demonstrated higher filling ability in the case of SC obturation compared to powder–liquid CSBS sealers [16]. Among the available premixed CSBS formulations, TotalFill^®^ BC Sealer (FKG Swiss Endo, Le Crêt-du-Locle, Switzerland) has been extensively investigated highlighting its hydrophilic nature, bioactivity and biocompatibility [17]. Furthermore, TotalFill^®^ BC Sealer can interact with dentine tissues promoting hydroxyapatite formation, with no shrinkage, ensuring a good sealing ability [18].

To the best of our knowledge, only a few studies assessed the influence of the CSBS placement technique on void occurrence [15,19,20]. This research topic demands further exploration aiming to better understand CSBS penetration and distribution into the root canal space during obturation and their potential related extrusion in the periapical area. In light of these considerations, the aim of the study was to assess the impact of the TotalFill^®^ BC Sealer insertion technique on the quality of the SC obturation by investigating the void occurrence and the sealer extrusion. The null hypotheses are that there are no significant differences in voids and extrusion between all the insertion techniques used.

## 2. Materials and Methods

### 2.1. Sample Selection

This study was approved by the Ethics Committee on the 12 November 2019 in Beirut, Lebanon (Laboratories of Saint Joseph University—Beirut, Lebanon), under the following ID: FMD-199/2019-241. From a pool of 500 first human mandibular premolars extracted for periodontal reasons, 36 premolars with one root canal (9 canals per group) were selected for this study following a power analysis for one-way ANOVA (4 groups) using G*Power software 3.1.9.7 for Windows (Heinrich Heine, Universität Düsseldorf, Düsseldorf, Germany). A power of 0.8, and an alpha level of 0.05 were considered, and an effect size of 0.6 was calculated based on a previous study [19].

**Figure 1:**
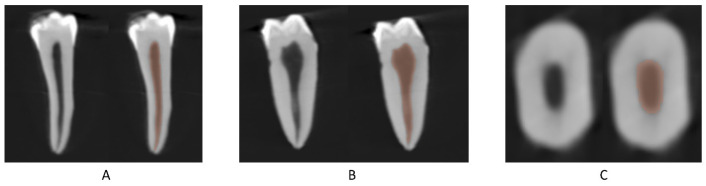
Frontal (**A**), cross-section (**B**) and axial (**C**) CBCT view of an initial premolar with and without the segmentation of the canal.

Inclusion criteria were defined after cone beam computed tomography (CBCT) imaging and analysis. Only well-developed single-rooted teeth with Vertucci type I configuration, straight root canal (curvature < 5 degrees) and with similar canal volume and initial apical diameter and cross-sectional canal shape, confirmed by CBCT’s software at the laboratories of Saint Joseph University in Beirut, were included in the present study (Figure 1). Teeth with previous endodontic treatment, cracks, caries or resorptions were excluded.

### 2.2. Root Canal Shaping and Cleaning

All experiments were carried out by the same experienced endodontist. Access cavity preparations on all premolars were performed using #802 and Endo Z burs (Dentsply, Maillefer, Ballaigues, Switzerland). Apical patency and glidepath were established manually using a size 10 K-file (VDW, Munich, Germany) and working length (WL) was acquired by moving back the instrument until reaching the major apical foramen minus 0.5 mm. Then, the crown of each premolar was cut in order to standardize the WL to 12 mm. All root canals were shaped with Reciproc R25^®^ (VDW, Munich, Germany) according to the manufacturer’s instructions. The instrument was used with 3 mm amplitude and its flutes were cleaned after 2 to 3 in–out movements. The sequence was repeated until carrying R25^®^ to WL. Apical patency was rechecked after root canal shaping. Apical gauging was carried out before obturation to insure that the apical diameter of each shaped canal was 0.25; this assured the standardization of all samples’ apical preparation after shaping. During shaping, root canal disinfection was performed using a total of 10 mL of NaOCl for every root canal. A final flush of ethylene diamine tetra acetic acid (17% EDTA) was performed by agitating the solution with Endoactivator (Dentsply, Sirona, Bensheim, Germany) for 1 min. Then, 3 mL of NaOCl were activated using Endoactivator for 1 min, followed by a rinse with 3 mL of the same solution. Finally, 3 mL of sterile water were applied. Root canals were then dried with paper points before obturation with no desiccation of the dentin walls [8].

Following this procedure, the apical third of each root was vertically embedded using modeling wax (Cavex, Haarlem, The Netherlands) then into acrylic resin (Technovit 4071, Hanau, Germany) in order to simulate the periodontal tissues.

### 2.3. Root Canal Obturation

For each root canal, the master-cone was adjusted and checked using a radiograph to make sure that it was reaching minus 0.5 mm from the WL. Then, these 36 premolars were randomly divided into 4 groups of 9 premolars each depending on TotalFill^®^ BC Sealer (FKG Swiss Endo, Le Crêt-du-Locle, Switzerland) insertion method, as follows:

Group A (*n* = 9): the sealer was placed in the coronal two-thirds of each root canal using the specific Total Fill^®^ tip introduced at 8 mm within the root canal. Then, the R25 gutta-percha cone (VDW, Munich, Germany) was slowly inserted until reaching the WL. The gutta-percha master cone was sectioned at the level of the coronal orifice and slightly condensed with a plugger vertically.

Group B (*n* = 9): the sealer was placed in the coronal two-thirds of each root canal using the specific Total Fill^®^ tip introduced at 8 mm within the root canal. Then, the sealer was activated using the medium sized EndoActivator tip (Dentsply Sirona, Bensheim, Germany) of 25/04 at minus 1 mm depth level, for 3 s, before slowly inserting the R25 gutta-percha cone until reaching WL. The gutta-percha master cone was sectioned at the level of the coronal orifice and slightly condensed with a plugger vertically.

Group C (*n* = 9): the sealer was placed in the coronal two-thirds of each root canal using the specific Total Fill^®^ tip introduced at 8 mm within the root canal. Then, the gutta-percha cone was slowly introduced into the root canal until reaching WL. While still holding the cone with a metallic tweezer, the ultrasounds (Start X #3) (Dentsply, Sirona, Bensheim, Germany) were applied on the latter for 3 s [21], in order to indirectly activate the sealer. The master-cone was then sectioned at the level of the coronal orifice and condensed slightly with a plugger vertically.

Group D (*n* = 9): positive control group. TotalFill^®^ was placed on the full surface of the last 12 mm of the gutta-percha cone from its tip. Then, the latter was slowly inserted inside the root canal until reaching WL. The master-cone was sectioned at the level of the coronal orifice and slightly condensed with a plugger vertically.

The access cavities were filled with a glass ionomer cement (Cavex Holland BV). Teeth were afterwards stored in an incubator at 37 °C and 100% humidity for 7 days. Then, a square of Plexiglas (2 × 2 cm) was bonded on the coronal side of the root to facilitate its subsequent positioning during the micro-computed tomography scanning.

### 2.4. Micro-Computed Tomography Scanning

The micro-CT Platform (EA2496, Hopkinton Mont-Rouge, “Paris-Descartes” laboratories, Paris Universités, France) was used to scan each root using micro-CT scanner (Quantum FX, Perkin Elmer Heath Sciences, Hopkinton, MA, USA). A 20 mm field of view was used to acquire 3D images with an isotropic resolution of 40 μm. The settings were as follows: 160 kV, 90 mA and 360 degrees scanning rotation.

### 2.5. Voids Measurements and Calculation

**Figure 2:**
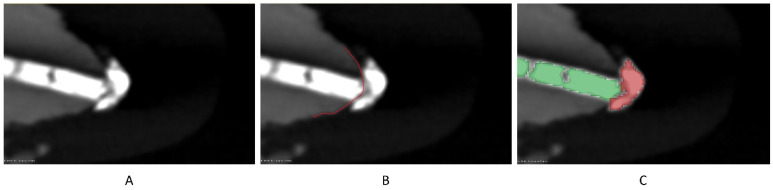
The distinction between the filling inside the canal (green) and the extrusion (red) (**C**) achieved by following the tooth contour (**B**) after micro CT scan (**A**).

**Figure 3:**
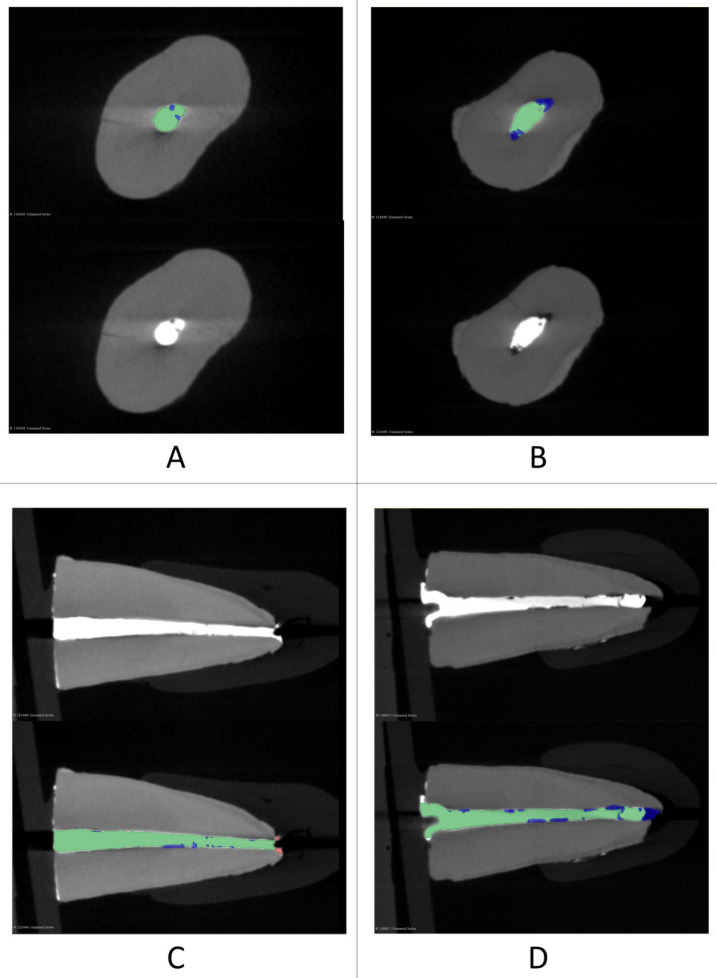
Two axial views (one tooth from each group: (**A**,**B**)) and two cross-sections (one tooth from each group: (**C**,**D**)) with and without the segmentation of the filling (green) and voids (blue).

The software 3D Slicer (version 5.2.2) was used to measure the filling material volume, the volume of the voids between the filling material and the tooth structure, and the volume of the extruded filling material. All evaluations were carried out blindly by the same experienced operator. The filling material was segmented automatically by adjusting the threshold to include all the radiopaque material, and then the extruded part was deleted manually (Figure 2). The extruded part was then segmented separately by using the same threshold, and the voids were manually segmented. All segmentations were exported to 3D models used by the software to calculate their volumes (VF for the volume of filling material, VV for the volume of the voids, and VE for the volume of the extruded filling material) (Figure 3). Then, the percentage of voids (V%) was calculated by dividing the VV by VV + VF. The 2 models were also split into 3 thirds to obtain 3 filling models and 3 void models for each one (apical, middle, and coronal of each root). The volumes of those models were used to calculate the percentage of the voids in each root canal third. All the internal voids that were completely surrounded by the radiopaque gutta-percha were not included in the segmentation, thus their volume was not calculated.

### 2.6. Statistical Analysis

Data were analyzed using IBM SPSS Statistics for Windows, v.26 (IBM Corp., Armonk, NY, USA). Means ± standard deviations and medians (interquartile ranges) were calculated and reported for the quantitative variables. The normality of distribution was assessed using the Shapiro–Wilk test, and since all outcome variables were not normally distributed, the Kruskal–Wallis test was used to compare independent means among groups. To compare dependent means within the same group and among root levels, the Friedman’s test was used. Tests were followed by the Bonferroni post hoc test for multiple pairwise comparisons. The level of significance was set at 5% and all tests were two-sided.

## 3. Results

Group B showed the lowest mean and median in terms of voids (Table 1) while group D showed the highest, with no statistically significant differences among the four groups (*p* > 0.05).

Within group A, the highest observed median was in the apical third and the lowest in the middle third (Table 2). However, no statistically significant differences were observed among root levels (*p* = 0.641). Within group B, the highest observed median was in the coronal third and the lowest in the middle third. However, no statistically significant differences were observed among root levels (*p* = 0.107). Within group C, the highest observed median was in the apical third and the lowest in the coronal third. However, no statistically significant differences were observed among root levels (*p* = 0.717). Within group D, the highest observed median was in the coronal third and the lowest in the apical third. However, no statistically significant differences were observed among root levels (*p* = 0.368). Regardless of groups, the differences among root levels were not statistically significant (*p* = 0.259). No statistically significant differences were observed among groups, for the apical, middle, and coronal voids’ percentages.

Group D showed the lowest value in terms of extruded volume while the highest value of extrusion was noted in group C with a statistically significant difference between the two groups (Table 3). No significant differences between groups A, B and D; A B and C groups were noted in terms of sealer extrusion.

**Figure 4:**
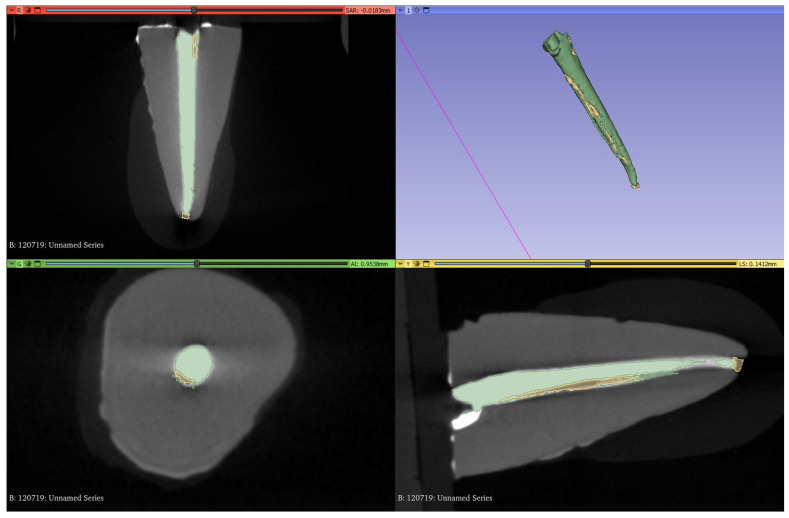
**The** 3D Slicer software showing one micro-CT scan of filling material (green) selected and calculated voids (yellow) and extruded volume.

Figure 4 shows the interface of the software which was used in the present study to understand the overall environment of the software in the selection and calculation of voids.

## 4. Discussion

The purpose of the present study was to evaluate the influence of different CSBS insertion techniques on the void occurrence and sealer extrusion, under a comparative micro-computed tomography analysis. In our study, the null hypothesis was accepted for void formation and rejected for sealer extrusion. In recent years, the advent of micro-CT has transformed the field of endodontics by providing unparalleled insights into the quality of obturation within the root canal system [22]. As a non-invasive technique, the process allows the detection of minor voids that are non-apparent in two-dimensional radiographs [23]. Unlike conventional radiography, micro-CT offers three-dimensional imaging and is considered very reliable nowadays, enabling a meticulous assessment of voids in intricate anatomical spaces and differentiating gutta-percha, endodontic sealers and internal or external voids [22,23]. In the present study, only open porosity, which is defined as voids occurring in the interface between dentin walls and gutta-percha/sealer (external voids) and combined voids between canal walls, gutta-percha and sealers were calculated [24] as they offer a potential pathway for microorganism growth and migration toward the periapical region [25,26]. Closed porosity is an isolated unfilled space within the sealer, which has much less or no potential for bacterial growth and migration [26,27]. Indeed, in our investigation, considering the difference and effect of each porosity on the clinical outcome, it was important to distinguish the location and type of voids for a more precise calculation. In terms of clinical relevance, it seemed rational to focus more on open porosity, which may contribute to endodontic failures [26,27].

The significance of achieving a hermetic seal in endodontics should not be underestimated. This implies the creation of a barrier against bacterial ingress and reinfection, which are critical factors for the long-term success of the root canal treatment [28]. The necessity of this seal arises from the intricate nature of the root canal anatomy, where complexities and irregularities can harbor bacteria, thereby necessitating a “technique-sensitive” obturation process [29]. In this context, the updated SC technique indicated with the use of CSBS might have inherent limitations especially when applied in oval root canals [12]. For this reason, mandibular premolars were selected in the current study for their oval root canals in the coronal and middle thirds, in order to simulate a realistic clinical scenario [29].

In the present study, it was shown that the sealer insertion technique may influence the formation of voids. Among the various techniques examined, direct sonic activation of the sealer using EndoActivator was found to be effective in minimizing voids. Even if the results showed no statistically significant differences between the different sealer placement techniques, the use of EndoActivator for 3 s (group B) demonstrated around 3 times fewer voids than the control group D, as the voids’ means varied between almost 3.5% for group B and 9.9% for group D. This sonic device is initially claimed to create agitation and a hydrodynamic activation of endodontic irrigants [30]. From the same concept, EndoActivator use after sealer placement may contribute to spread the latter laterally and vertically, ensuring a more uniform CSBS penetration/distribution within the root canal space.

Similarly, indirect activation of the master cone while it was held with tweezers yielded favorable outcomes, highlighting the importance of proper placement and the indirect ultrasonic activation of CSBS. This is in accordance with the results of one previous micro-CT study, showing better root canal filling quality when the master cone and sealer together inside the root canal space were under indirect ultrasonic activation, compared to the conventional SC technique with no activation [21]. A lower score of voids following CSBS ultrasonic activation compared to the control group (no activation) was also noted in a recent study, based on micro-CT [31]. The same study also showed more voids when the assessment was performed under a stereomicroscopic examination of sectioned samples [31]. When using ultrasonic activation, gentle vibration is recommended rather than excessive energy which may accelerate void formation and negatively affect the obturation quality [32]. To date, no guidelines are yet available regarding sonic or ultrasonic sealer activation. For this reason, further research is needed to clarify the potential benefits of these approaches on void scores and clinical outcome. None of the techniques used in the present study showed gap-free areas along the gutta-percha/sealer interface, which is in accordance with the results of recent studies [22,33].

Notably, the control group D, where the sealer was applied on the master cone before insertion, and group A where sealer was injected in the coronal two-thirds with no activation, exhibited the highest mean voids. Even though there were no statistically significant differences noted between the groups, it remains important to mention that the highest mean of voids in both groups A and D may be attributed to the inability of the master cone insertion alone to properly distribute the CSBS within the root canal space [34].

Interestingly, all techniques showed no statistically significant differences between the apical, coronal, and middle thirds in terms of voids. Moreover, regardless of groups, the difference among root levels was not statistically significant. This uniformity is attributed to the meticulous standardization of the samples and the precise apical fitting of the matching Reciproc^®^ R25 master cone within the prepared root canals. The use of CBCT scanning and standardized sample volumes further enhanced the comparability of the results. This is in contradiction with the results of other studies, showing that coronal and middle thirds demonstrated higher means of voids compared to the apical third [22,34]. This could be explained by the fact that the roots in the present study were cut at 12 mm length, indicating that the large and oval part of the root canal in the coronal third of each sample was removed.

In the present study, sealer extrusion was significantly lower in the control group compared to the group using indirect ultrasonic activation of CSBS. This underscores the fact that ultrasonic activation may lead to a higher risk of sealer extrusion in the periapical area. More studies are required to better understand if the duration of ultrasonic activation may impact sealer extrusion. Nevertheless, perfect apical control is impossible to perform especially when maintaining the apical patency throughout the endodontic procedure and in the case of extrusion, the fate of sealers will depend on their solubility in periapical tissue fluids and their susceptibility to phagocytosis [35]. It has been suggested that lower healing/success rates in the case of sealer extrusion were related to its cytotoxicity, chemical composition and solubility [35,36]. Moreover, over-fillings might dissolve in periapical fluids and then can be wrapped by fibrous tissues [37]. A histological exam revealed that an inflammatory response may occur in periapical tissues shortly after sealer extrusion; nonetheless, toxicity vanishes and the reaction stops after the sealers set [37,38]. A recent clinical study investigated iRoot SP extrusion, which has the same composition as TotalFill^®^ BC Sealer. The results showed that iRoot SP extrusion had no adverse effect on the outcome of root canal treatment. Notably, inadvertent CSBS extrusion/surplus should not be clinically relevant especially considering their improved biocompatibility and bioactivity compared to conventional sealers [37].

One of the study’s limitations was the number of samples (*n* = 9 per group), even though this number was obtained by sample size calculation; a larger number of roots may have given clearer and more powerful results. A second limitation is the fact that this study only evaluated Total Fill^®^ BC Sealer. Including more types of CSBS would provide a better understanding of the differences in their clinical performance in terms of penetration/distribution into the root canal space depending on the placement technique. Another limitation was the measurement of the void volume by only one analytical method, i.e., the micro-CT technique. Volumetric measurements performed using micro-CT analysis should be carefully interpreted, as factors such as voxel size, image-processing software, and material radiopacity may influence the measurements [39].

## 5. Conclusions

In conclusion, the present study demonstrated that the sealer insertion technique could impact the ratio of void occurrences following single-cone obturation. In fact, using direct sonic and indirect ultrasonic activation showed the most promising results despite the absence of statistically significant differences among the groups. Ultrasonic activation, however, may lead to more sealer extrusion compared to the control group.

## Figures and Tables

**Table 1:** Comparison of voids’ percentage among groups regardless of root levels (*n* = 36).

	Total Voids’ Percentage			
	Mean ± SD	Median (Q1–Q3)	Range *(Minimum–Maximum)*	*p*-Value
**Group A (*n* = 9)**	6.759 ± 6.539	3.431 (1.818–12.068)	0.686–18.864	0.066
**Group B (*n* = 9)**	3.546 ± 2.849	2.662 (0.999–6.148)	0.012–7.883	
**Group C (*n* = 9)**	4.284 ± 3.994	3.084 (0.997–7.647)	0.648–12.187	
**Group D (*n* = 9)**	9.953 ± 6.402	7.898 (4.634–16.303)	2.182–20.646	

SD = standard deviation; Q1 = first quartile; Q3 = third quartile.

**Table 2:** Comparisons of voids’ percentage according to root levels and groups.

	Voids’ Percentage			
	Apical Third	Middle Third	Coronal Third	*p*-Value
**Group A**				
Mean ± SD	12.304 ± 17.250	3.609 ± 3.886	7.479 ± 8.703	
Median (Q1–Q3)	5.217 (0.017–23.590)	1.596 (0.674–7.963)	3.456 (1.944–13.010)	0.641
Range (min–max)	0.000–47.826	0.000–9.854	0.781–25.907	
**Group B**				
Mean ± SD	2.397 ± 3.436	1.408 ± 2.239	4.506 ± 4.148	
Median (Q1–Q3)	0.368 (0.000–5.205)	0.135 (0.004–2.526)	4.330 (0.261–7.236)	0.107
Range (min–max)	0.000–8.955	0.000–6.466	0.022–12.212	
**Group C**				
Mean ± SD	4.986 ± 4.315	4.276 ± 4.071	3.959 ± 5.217	
Median (Q1–Q3)	5.050 (0.910–7.732)	3.404 (1.091–7.242)	2.247 (0.431–6.448)	0.717
Range (min–max)	0.219–13.415	0.000–12.371	0.000–15.636	
**Group D**				
Mean ± SD	8.028 ± 9.527	9.138 ± 9.508	10.640 ± 9.548	
Median (Q1–Q3)	2.083 (1.157–15.983)	6.513 (1.894–16.832)	11.196 (2.510–15.062)	0.368
Range (min–max)	0.000–26.364	0.000–27.157	0.904–30.995	
***p*** **-value**	0.357	0.063	0.194	
**Total**				
Mean ± SD	6.929 ± 10.474	4.608 ± 6.103	6.646 ± 7.458	
Median (Q1–Q3)	3.195 (0.246–8.594)	1.934 (0.568–6.861)	3.556 (1.110–10.707)	0.259
Range (min–max)	0.000–47.826	0.000–27.157	0.000–30.995	

SD = standard deviation; Q1 = first quartile; Q3 = third quartile; min = minimum, max = maximum.

**Table 3:** Comparison of extruded filling volume (in mm^3^) among groups (*n* = 36).

	Extruded Filling Volume (mm^3^)			
	Mean ± SD	Median(Q1–Q3)	Range *(Minimum–Maximum)*	*p*-Value
**Group A (*n* = 9)**	0.190 ± 0.294	0.070 (0.005–0.300) ^AB^	0.000–0.900	0.044 *
**Group B (*n* = 9)**	0.173 ± 0.256	0.050 (0.000–0.290) ^AB^	0.000–0.760	
**Group C (*n* = 9)**	0.668 ± 1.000	0.110 (0.000–1.360) ^A^	0.000–2.860	
**Group D (*n* = 9)**	0.014 ± 0.043	0.000 (0.000–0.000) ^B^	0.000–0.130	

SD = standard deviation; Q1 = first quartile; Q3 = third quartile. * *p* < 0.05. Different uppercase superscript letters indicate statistically significant differences between groups.

## Data Availability

The data presented in this study are available on request from the last author.
